# Implementing social prescribing in primary care in areas of high socioeconomic deprivation: process evaluation of the ‘Deep End’ community Links Worker Programme

**DOI:** 10.3399/BJGP.2020.1153

**Published:** 2021-09-21

**Authors:** Nai Rui Chng, Katie Hawkins, Bridie Fitzpatrick, Catherine A O’Donnell, Mhairi Mackenzie, Sally Wyke, Stewart W Mercer

**Affiliations:** Institute of Health and Wellbeing, College of Social Sciences, University of Glasgow, Glasgow.; Usher Institute, College of Medicine and Veterinary Medicine, University of Edinburgh, Edinburgh.; Institute of Health and Wellbeing, College of Medical, Veterinary and Life Sciences, University of Glasgow, Glasgow.; Institute of Health and Wellbeing, College of Medical, Veterinary and Life Sciences, University of Glasgow, Glasgow.; Institute of Health and Wellbeing, College of Social Sciences, University of Glasgow, Glasgow.; Institute of Health and Wellbeing, College of Social Sciences, University of Glasgow, Glasgow.; Usher Institute, College of Medicine and Veterinary Medicine, University of Edinburgh, Edinburgh.

**Keywords:** community link workers, complex interventions, general practice, healthcare inequalities, primary care, social prescribing

## Abstract

**Background:**

Social prescribing involving primary care-based ‘link workers’ is a key UK health policy that aims to reduce health inequalities. However, the process of implementation of the link worker approach has received little attention despite this being central to the desired impact and outcomes.

**Aim:**

To explore the implementation process of such an approach in practice.

**Design and setting:**

Qualitative process evaluation of the ‘Deep End’ Links Worker Programme (LWP) over a 2-year period, in seven general practices in deprived areas of Glasgow.

**Method:**

The study used thematic analysis to identify the extent of LWP integration in each practice and the key factors associated with implementation. Analysis was informed by normalisation process theory (NPT).

**Results:**

Only three of the seven practices fully integrated the LWP into routine practice within 2 years, based on the NPT constructs of coherence, cognitive participation, and collective action. Compared with ‘partially integrated practices’, ‘fully integrated practices’ had better shared understanding of the programme among staff, higher staff engagement with the LWP, and were implementing all aspects of the LWP at patient, practice, and community levels of intervention. Successful implementation was associated with GP buy-in, collaborative leadership, good team dynamics, link worker support, and the absence of competing innovations.

**Conclusion:**

Even in a well-resourced government-funded programme, the majority of practices involved had not fully integrated the LWP within the first 2 years. Implementing social prescribing and link workers within primary care at scale is unlikely to be a ‘quick fix’ for mitigating health inequalities in deprived areas.

## INTRODUCTION

Health inequalities continue to grow globally and in the UK.^1^^–^3 People living in areas of high socioeconomic deprivation have multiple health problems that are caused or exacerbated by complex socioeconomic factors.^4^ Supporting patients with such complex multimorbidity is a challenge for primary care.^5^^–^7 Social prescribing is widely promoted as a way of reducing health inequalities by better supporting people living in deprived areas.^8^^–^12 Its potential to do any more than mitigate the effects of the social determinants of health, however, has been questioned because, like other health sector interventions, it individualises the problem of inequalities and targets individual behaviours as the main solution.^13^

Social prescribing aims to link patients seen in primary care with local community resources and provides GPs with a route of referral for non-medical support that can be used alongside or instead of existing medical treatments.^11^^,^^12^^,^^14^^,^^15^ It can be facilitated by community link practitioners (CLPs) within primary care, who can spend time with referred patients to understand their situation and needs, and can then suggest appropriate community resources based on their in-depth local knowledge.^15^^–^17

Social prescribing using primary care-based link workers is increasingly promoted across the four nations of the UK.^18^^–^22 Despite a high level of support from policymakers, high-quality evidence on the effectiveness of the link worker model of social prescribing is scarce.^14^^,^^17^^,^^23^^–^25 There is also limited knowledge on how best to implement the link worker approach so that link workers can be embedded and integrated in primary care settings to maximise their effectiveness and sustainability.^26^^,^^27^

The Scottish Government is promoting social prescribing, as a way of reducing or mitigating health inequalities, with a pledge to roll out 250 link workers nationally by the end of the parliament in 2021. It preceded this by piloting the ‘Deep End’ Links Worker Programme (LWP), which targeted GPs based in practices serving some of the most deprived patients in Glasgow.^28^ They also funded a quasi-experimental external evaluation, in which the practice of the LWP’s clinical lead was assigned as an intervention practice. Other practices in deprived areas volunteered to take part and were randomised either to deliver the LWP, as intervention practices, or to continue with usual care, as comparison practices.^29^

The authors of this study have previously reported on quantitative patient-reported outcomes, comparing the intervention with comparison practices.^25^ Intention-to-treat analyses found no differences between intervention and comparison practices for any outcome. Subgroup analysis showed that patients who saw a CLP on ≥3 occasions (45% of those referred) had significant improvements in quality of life, depression, anxiety, and exercise levels. This same author group also reported on the views of CLPs and community organisations on the sustainability of community organisations.^30^ That study found positive experiences of collaborative working, particularly the CLPs’ ability to act as patients’ case manager, and as a bridge between organisations, but there were also challenges to the capacity and funding for community organisations in the context of austerity.

**Table:** How this fits in

Social prescribing using primary care-based link workers is increasingly promoted across the four nations of the UK, and elsewhere in the world, as a way of reducing health inequalities by better supporting people living in deprived areas. The evidence base of effectiveness is, however, limited and there is very little information on how best to successfully implement a link worker approach in practice. This study reports on a process evaluation of the ‘Deep End’ Links Worker Programme (LWP) over a 2-year period, in seven general practices in deprived areas of Glasgow. Despite the programme being well funded and well supported, the majority of practices involved had not fully integrated the LWP within the first 2 years. Implementing social prescribing and link workers within primary care at scale is unlikely to be a ‘quick fix’ for mitigating health inequalities in deprived areas.

In this study the implementation of the LWP in the seven intervention practices, the extent to which the programme was integrated into routine practice, and the factors that helped or hindered this are explored. A recognised implementation theory — normalisation process theory (NPT) — is used to enhance understanding of factors that supported, or hindered, implementation and sustainability.

## METHOD

### Design and setting

A qualitative process evaluation with staff in seven practices taking part in the LWP was conducted. A qualitative approach was chosen because it allows good insight into implementation processes and is highly suitable for process evaluations.^31^ Practices in Glasgow qualified to be part of the Deep End project if they were within the 100 most deprived practices in Scotland, based on the percentage of registered patients in practices living in the 15% most deprived postcodes in Scotland (see https://www.gla.ac.uk/researchinstitutes/healthwellbeing/research/generalpractice/deepend). Practice staff who participated in the evaluation provided written informed consent.

This process evaluation was part of a mixed-methods evaluation of the LWP.^26^^,^^29^

### Intervention

Each intervention practice had a full-time salaried CLP appointed, who was employed by a Scottish Government-funded third-sector organisation (the Health and Social Care Alliance Scotland, https://www.alliance-scotland.org.uk) but based in the practice. The CLPs were selected by the programme manager and clinical lead, who also made the final decision on which practice to assign each CLP. CLPs assumed posts within each practice on 2 April 2014.^32^

Intervention practices were also provided with a practice development fund of £35 000, around 80% of which was used for creating more time, particularly clinical time for GPs (and the practice nurse in one practice) to have longer consultations with patients. Practices also invested to free-up receptionist time, by, for example, hiring another receptionist or purchasing self-check-in systems.

Finally, intervention practices were also offered additional programme management support by the CLPs’ employing organisation. Support included 1) an experienced programme director, whose work included producing detailed records of learning; 2) a senior community links manager, responsible for establishing protocols and policies for CLP work and line managing the CLPs; 3) a learning and evaluation officer, responsible for establishing local protocols for programme monitoring (independent of the evaluation conducted by the research team); 4) administrative staff; and 5) a clinical lead.

The LWP was expected to operate at three levels: patient, practice, and community (see Supplementary Box S1):

- at the patient level, practices were to set up referral systems so that GPs and practice nurses could refer patients who they thought would benefit from engagement with community resources to the CLP for one-to-one work;
- at the practice level, CLPs were also expected to act as ‘agents of change’, promoting the ethos of social prescribing among all staff by, for example, enabling activities to support staff wellbeing, activities to develop shared learning, and awareness about community resources, gathering ‘intelligence’ about local resources and solving problems through the redeployment of staff; and
- at the community level, CLPs were expected to build networks and cultivate relationships with local community organisations, develop referral pathways and multiagency resolution of problems, and organise shared learning events to consolidate new and existing community linkages.

### Process evaluation

This evaluation was guided by NPT, which argues that successful integration of new innovations requires four kinds of work when attempting to implement a new practice (Box 1).^34^^,^^35^

**Box 1.** NPT constructs adapted to the LWP

**Core construct**	**Explanation^33^**	**Questions in terms of LWP**
Coherence	The sense-making work that people do individually and collectively when they are faced with the problem of operationalising some set of practices.	Do people understand LWP and see it as different from other/previous ways of working?
Cognitive participation	The relational work that people do to build and sustain a community of practice around a new technology or complex intervention.	Are people willing and able to engage with one another to carry out the LWP?
Collective action	The operational work that people do to enact a set of practices, whether these represent a new technology or complex healthcare intervention.	What do people do to carry out the LWP and how? What additional resources and support are required?
Reflexive monitoring	The appraisal work that people do to assess and understand the ways that a new set of practices affect them and others around them.	How do people know if the LWP is effective and can they modify it?

*LWP = Links Worker Programme. NPT = normalisation process theory.*

### Participants

Participants were practice staff with responsibility for leading the LWP (lead GPs, CLPs, and practice managers) and community organisation workers identified by CLPs.

### Data collection

Four methods of data collection were used — focus group discussions, email surveys, in-depth interviews mid-implementation, and in-depth end-of-evaluation interviews in four phases (Box 2 provides an overview). Data from focus group discussions were used to produce LWP’s overall theory of change (see Supplementary Box S1). To collect information longitudinally and understand how practice views and actions changed as the LWP developed, email surveys (phase [P]2 and P4) and two waves of in-depth interviews (P2 and P3) were used. Staff interviews and focus groups lasted 40–90 min, were audiorecorded, and transcribed verbatim.

**Box 2.** Data-collection methods, participants, and time points

**Data-collection type**	**Participant types and numbers**	**Purpose**	**Phase**
Focus group discussion in each practice	Lead GPs, CLPs, and PMs. In some group discussions, other staff members (other GPs and practice nurses) were also invited by PMs or GPs if they were felt to have been particularly involved. Wherever possible a community organisation worker, identified by the CLP, was also invited. Seven sessions; 31 participants	Views of the LWP, its aims, how it was being implemented; identification of the underlying mechanisms of action.	P1 (Nov 2014 to Jan 2015) Early implementation phase
Email survey to staff in each practice	Lead GPs, CLPs, PMs, a reception/support staff member chosen by the PM, practice/district nurse, and staff from two different community organisations identified by the CLP. P2 — 44 sent; 38 replied; P4 — 30 sent; 19 replied	Open-ended questions to elicit information on any changes in how the LWP was implemented and other changes in local context.	P2 (Jun–Oct 2015) Mid-implementation phase P4 (Jun–Oct 2016) Final implementation phase
In-depth interview with lead staff in each practice	Lead GPs and CLPs. 14 interviews	To elicit more in-depth information on day-to-day LWP implementation, what worked well, what less well, and why.	P2 Mid-implementation phase
End-of-evaluation interview with lead staff in each practice	Lead GPs, CLPs, and PMs. 19 interviews	To elicit views on the success of the LWP, what worked well, what less well, and why.	P3 (Jan–Feb 2016) End-of-evaluation phase

*CLP = community link practitioner. LWP = Links Worker Programme. P = phase. PM = practice manager.*

### Data analysis

Data analysis used the framework approach supported by NVivo (version 10).^36^ The core analysis team supported the first author to develop and apply a thematic index. The index allowed coding to the four main NPT constructs as well as to descriptive explanations of the work done at patient, practice, and community levels, and this was applied across all datasets. The framework approach allowed the construction of practice case studies and comparison between them by charting all data sources within a matrix that was organised by practice. Practices were given a pseudonym and participants labelled by the initials of the type of participant (GP, CLP, and so on) within each practice.

The core team consisted of academics, with extensive experience of qualitative research: a medical sociologist, a senior health services researcher (also with experience of NPT), a social scientist (with experience of theory of change), and a clinical primary care academic. A postdoctoral political scientist (the first author) with experience of qualitative research in non-healthcare settings led the data analysis with support from the core team.

The reporting of this study here conforms to the Consolidated Criteria for Reporting Qualitative Research (COREQ) (see Supplementary Box S2).^37^ The COREQ checklist is an attempt at complete and transparent reporting, and also indirectly improves the rigour, comprehensiveness, and credibility of the study. The study was registered prospectively with International Standard Randomised Controlled Trials (ISRCT): ISRCTN80842457 and the protocol published.^29^

## RESULTS

### Differentiating two types of LWP practices using NPT

The framework analysis identified variation between practices in the implementation and integration of the LWP, which clustered into two distinct groups: fully integrated practices (FIPs), which included three of the seven practices (‘Magenta’, ‘Ochre’, and ‘Cyan’), and partially integrated practices (PIPs), which were the remaining four (‘Crimson’, ‘Cobalt’, ‘Olive’, and ‘Amber’).

The two types of practices did not differ in the number of registered patients on their lists (FIPs mean 4009 [range 2244–5130] versus PIPs mean 4349 [range 2549 to 5946]) nor in the number of patients who were black and minority ethnicity (FIPs mean 7.3%; PIPs mean 7.8%). However, there were fewer training practices in FIPs (one out of three) than PIPs (three out of four).

In FIPs, all aspects of the LWP were implemented and integrated into ways of working so that CLPs were empowered to undertake all aspects of their role — patient support, practice development, and community networking. In PIPs, by the end of the evaluation CLPs were enabled to undertake only some aspects of the LWP. In particular, although CLPs in PIPs did work directly with patients after referral, the practice development and community networking aspects of their work were much less well supported.

For example, in the early stages of the evaluation the authors saw that all practices had tried some activities to enhance staff wellbeing, had explored information systems about the availability of community organisations, and had organised some shared learning sessions for all staff. However, by the end of the evaluation, only FIPs continued these activities. A GP in a FIP reported the benefits of making time for shared learning within the practice:

*‘And I think the simple fact of having one afternoon a week where we go off site and we just sit and talk to each other, and have a coffee together, and interact in a more human way, it’s had a real change in the whole dynamic of the practice …’*

(Magenta GP, FIP, end-of-evaluation interview, P3)

This view contrasts with that of a CLP in a PIP:

*‘Yes, there’s a good rapport and the staff, you know, the administrative staff go out and things. But there is a disconnect between admin staff, and the GP staff. The admin staff as well don’t get team meetings. They don’t get opportunities to come together as a team and share information so communication sometimes can be a bit poor at different times.’*

(Cobalt CLP, PIP, end-of-evaluation interview, P3)

The difference between practices was also apparent in relation to community networking activities. Only in FIPs were CLPs enabled to be proactive and strategic, by, for example, making time each week to interact with staff in community organisations, and facilitating links between community organisations and staff in the practice. These activities were highly valued by the CLPs in FIPs:

*‘I sit on steering groups in the health centre. Sit on the arts and environmental steering group which is about the health centre and how it’s linking in with regards to arts and, like, so … Then I sit on the community-orientated primary care group, which is across the whole health centre … and it’s about, obviously, community-orientated primary care, linking them in, getting an awareness of what’s going on in the local area.’*

(Ochre CLP, FIP, in-depth interview, P3)

In PIPs, however, CLPs reported a more reactive approach to community networking. They were not able to make the time to develop, on an ongoing basis, a more strategic view of what was locally available and needed for different groups in the community. They often reported regret at being unable to do more proactively. For example, the CLP in Cobalt practice felt that she was not doing enough:

*‘I would like to be more, I think I would like to be more proactive. Whether that’s possible, like instead of someone coming to me, and me having a conversation with someone and then saying, “Right, OK, let’s look at what’s out there.” What I would quite like to do is to be able to go out and walk around* [place name] *, or* [place name] *, or whatever.’*

(Cobalt CLP, PIP, end-of-evaluation interview, P3)

Drawing on NPT, Box 3 shows that, compared with staff in PIPs, staff in FIPs had better and more shared understanding of the LWP (coherence), were more likely to engage (cognitive participation), and more likely to work to implement the LWP (collective action). Reflexive monitoring of progress with the LWP, however, was underdeveloped in both types of practice (Box 3). Supplementary Box S3 provides extracts from staff in all seven practices.

**Box 3.** Comparison of the implementation of the LWP in fully and partially integrated practices based on NPT

**NPT construct**	**FIPs (three practices)**	**PIPs (four practices)**
Coherence: understanding of the LWP	Core leadership (GP, CLP, and PM) share understanding of the LWP and how they want it to work. For example, in Cyan practice, the CLP clearly identified why they expected LWP to work: *‘It’s* […] *trying to build-up knowledge of what is actually out there … So* […] *part of the programme is for myself to work one to one with people but for the whole practice to be more aware of what* [community organisations are] *actually around that maybe would support patients and I guess* […] *try and develop relationships with some of* [the staff in] *these resources.’* (Cyan CLP, focus group discussion, P1)	Core leadership (GP, CLP, and PM) do not share understanding of the LWP and how they want it to work. For example, in Cobalt practice, the CLP reported that, although she understood there were three aspects of her job, the lead GP thought the LWP was about being able to refer patients to her (and nothing else): *‘We kept getting told* [in training and support] *there’s three parts to this job, there’s your one-to-one work* [with patients]*, there’s community building, and there’s practice development. Well if that’s the case, you need to have scope to do all three. You know, and it can’t always be about patients, patients, and patients.’* (Cobalt CLP, in-depth interview, P2)
Cognitive participation: staff willing and able to engage with one another to carry out the LWP	Staff engage with each other on the LWP in both formal (meetings and shared learning activities) and informal (over coffee) settings. For example, in Magenta practice, the CLP was able to work effectively with the whole team to develop the LWP ethos: *‘In terms of attitude, there’s been a huge shift from medical to holistic, where the GPs are seeing beyond the medical presentation and looking at the root core cause, knowing I think, knowing that they have someone to back up their findings, where before they wouldn’t ask certain questions because they couldn’t do anything about it.’* (Magenta CLP, in-depth interview, P2)	Less staff engagement in formal settings (meetings and shared learning activities) and more in informal (over coffee) settings. For example, in Olive practice, the CLP explains that practice staff have not engaged much and that there are fundamental differences in understanding of the LWP and the CLP’s role: *‘It’s taken our district nurse a year to understand what it is I do and we still sometimes clash on approach and understandings and what that’s about. So, you know, but it’s taken her a year to get to grips. She spent the first year telling everybody I was a psychologist, do you know what I mean?’* (Olive CLP, in-depth interview, P2)
Collective action: what staff in practices did to deliver the LWP (focus on work of CLP)	CLP’s role in practice development unconstrained and work balanced across patient support, practice development, and community networking. For example, in Ochre practice, the CLP explains that the whole practice team now has relationships with community organisations and are confident to liaise on behalf of patients: *‘So say … If the receptionist had booked an appointment at one of the community organisations … They’d* [the community organisation] *be quite happy if the receptionist calls* [to enquire about a referred person] *— they can say “Oh, that person didn’t turn up”, and the receptionist might ring the person up and go “Oh … you know, they said that you didn’t turn up. Do you want any support?”… and then they might get referred to me so I can support them, and it’s kinda definitely linking things up. So … Yeah. And I do think it’s really good that the practice staff feel more confident in referring to community organisations.’* (Ochre CLP, in-depth interview, P2)	CLP’s role in practice development constrained; more focus on one-to-one patient support than other activities. For example, in Amber practice, changes in CLP staff made community networking, a central aspect of the LWP, difficult to maintain. In the first email survey (P2) both the PM and GP noted this. Asked what had been difficult to action: *‘Network building — Too time consuming to allow me to do this. The networking done by our CLP is very helpful. Knowing what resources there* *are out in the community benefits the team to confidently inform a patient* *about a service.’* (Amber PM, email survey, P2) *‘Network building — this has been slower to achieve than I first anticipated.’* (Amber GP, email survey, P2)
Reflexive monitoring: how staff knew if LWP was effective	Reflexive modelling was underdeveloped in both FIPs and PIPs. There was no formal monitoring of LWP implementation in any practice. Informal monitoring, based on how people thought ‘things were going’, was used instead. For example, in Magenta practice, there is not a system to capture information about what is happening in the programme in terms of all the activities and tasks. There is a difficulty with recording activities and monitoring: *‘It’s quite difficult to get it all in because there’s a lot happening.* [laughs] *And it’s quite hard to sit down and sort of capture all the different elements that are happening to us.’* (Magenta GP, in-depth interview, P2). But staff can provide feedback at other times (for example, during protected learning times made possible by the LWP), and staff used impressions when asked ‘how they know if the LWP is working’. The Magenta GP said, for example, that they think that patients now know something about how the practice has changed: *‘I get the impression that patients have felt there’s a different feel about the practice.* [And noted that] *complaints have dropped dramatically.’* (Magenta GP, end-of-evaluation interview, P3)	

*CLP = community link practitioner. FIP = fully integrated practice. LWP = Links Worker Programme. NPT = normalisation process theory. P = phase. PIP = partially integrated practice. PM = practice manager.*

### Factors influencing the implementation process in the two types of practices

Cross-case comparison between FIPs and PIPs suggested four factors influenced whether or how the LWP was implemented: leadership, team relationships, continuity of CLP support, and the influence of other ongoing interventions.

In FIPs the leadership of the LWP was shared collectively between GPs, CLPs, and practice managers, there were empowering team relationships, continuity of CLP support (or transitions managed well), and no influence from other ongoing innovations. PIPs, on the other hand, had less collective leadership, more challenging team relationships, interrupted CLP support, and in one practice may have been distracted by another ongoing innovation on integration of health and social care.

Data extracts in Box 4 illustrate the findings of the analysis. Supplementary Box S4 provides data extracts from staff in all seven practices.

**Box 4.** Examples of how the contextual features of leadership, team relationships, continuity of CLPs’ support, and other innovations influenced LWP integration

**Leadership** was a key factor in how practices implemented LWP. In FIPs, leadership over the LWP was shared among key members of staff — the lead GP, CLP, and PM. A Magenta GP, for example, reflected that others in the practice were also taking on responsibilities: *‘I am continuing to provide leadership but have been pleased to see the wider team taking on roles and for activities such as the learning times to be embedded now in practice activities.’* (Magenta GP, email survey 2, P4)Compared with FIPs, leadership over LWP in PIPs was not as well shared and connected, as indirectly described by the GP of Crimson practice: *‘The programme was designed that they were basically dropped in with no structure, and I totally understand why that was done but it wasn’t easy. That was not easy, either for the Links worker or for us, to create a job from nothing.’* (Crimson GP, in-depth interview, P2)Creating the structure for a new programme to work is the responsibility of practice and LWP leadership, and it appeared to be challenged in Crimson practice. **Team relationships** in FIPs were more enabling and positive, which helped with the implementation of the programme, as suggested by the PM in Ochre practice: *‘We’ve done a few team-building events. And I think the positivity from that has been great. I mean, there’s definitely everybody, you know, you know, they feel, everybody feels appreciated.’* (Ochre PM, end-of-evaluation interview, P3) Team relationships in PIPs on the other hand seemed more challenging and, according to the PM of Olive practice, had a negative impact on programme implementation: *‘Practice staff seem to no longer be interested in the project, relationships seem to have broken down, and apart from the clinical staff there is little or no interest in the project at the moment.’* (Olive PM, email survey 2, P4) Not all practices experienced disruption of CLP support. However, FIPs that had turnovers in CLP staff appeared to have **managed CLP support** disruption well. For example, the incoming CLP of Cyan practice was able to ‘shadow’ his predecessor in a handover process, thus ensuring a smoother transition: *‘And then* [Outgoing CLP] *would brief me on what he’d already done with them* [patients] *and then we would have a meeting in the GP service clinic with some of the participants and then* [Outgoing CLP] *would kind of brief me again on where he sees the process going with these participants. So it was a bit of a handover process with some people.’* (Cyan CLP, in-depth interview, P2) PIPs managed disruption to CLP support less well. Amber practice, for example, was slowed in its LWP implementation when its CLP went on leave: *‘Our CLP is off … As yet we have no idea of when her return will be. We have cover once a week for patient referrals; however, this has changed our momentum with certain capacities.’* (Amber PM, email survey 1, P2) Not all practices had other ongoing innovations. Two practices that did were, however, PIPs, and the **influence of other ongoing interventions** appeared to have affected leadership and team relationships. While multiple ongoing interventions in the same setting may not necessarily be a negative factor, in the case of Cobalt practice, for example, it hindered the implementation of LWP because the GP and CLP did not share the same view on how the different interventions might work together: *‘So there’s a wee bit of like when you mention things, he’ll be like, “Oh that’ll be great for the* [other project] *Project.” And you’re like that, “No … that’s not the* [other project] *– this is the Links programme.” So yeah, so he has clear ideas in some ways, yeah, he probably does have clear ideas what he wants.’* (Cobalt CLP, in-depth interview, P2)

*CLP = community link practitioner. FIP = fully integrated practice. LWP = Links Worker Programme. P = phase. PIP = partially integrated practice. PM = practice manager.*

## DISCUSSION

### Summary

A longitudinal qualitative process evaluation of a well-funded government programme that aimed to embed social prescribing in primary care practices through the use of CLPs was conducted. Over a 2-year period, only three of seven practices fully implemented the programme as planned. Practices that fully integrated the LWP had a better shared understanding of the programme, higher staff engagement, and implemented the LWP at all three of its intended levels of impact (patient, practice, and community). Successful implementation was influenced by leadership, team relationships, how practices dealt with disrupted CLP support, and how practices dealt with other ongoing interventions in and around the practice. The two practice types did not differ in terms of their list size or ethnicity of patients, but there were more PIPs with training practice status compared with FIPs. Training practices are generally more innovative than non-training practices, which would not explain these findings.^38^

### Strengths and limitations

This study is based on a longitudinal qualitative analysis from a process evaluation involving a wide range of key stakeholders. Unlike most qualitative evaluations of social prescribing schemes that focus on the perspective of the service users,^39^ the current study highlights the role of the CLPs and the context in which they work. The work was also part of a broader, well-funded, and government-supported evaluation from which the analyses here were derived.^40^

A limitation was the relatively small number of practices who received the intervention and the limited timeframe for evaluation. Embedding new innovations in primary care can take many years,^41^ and the authors cannot say if the PIPs would become FIPs in the fullness of time or if the interventions would continue to be sustained in the FIPs.

### Comparison with existing literature

Much of the existing literature on social prescribing focuses on whether it is effective or not in terms of patient outcomes, including its capacity to contribute to the reduction of deep-seated inequalities.^13^^,^^14^^,^^17^^,^^42^ However, there is both limited evidence of effectiveness (and cost-effectiveness) and a limited number of high-quality quantitative studies with suitable control groups.^18^^–^22^,^^25^ There is also a significant knowledge gap regarding the process of implementation.^26^^,^^31^

A recent systematic review of factors facilitating implementation and delivery of social prescribing services in UK primary care found only eight relevant studies.^27^ The current study therefore adds to the limited literature on implementation of social prescribing services in primary care and the findings are broadly consistent with the conclusions of the systematic review.^27^ In addition, the view of the authors of this current study is that other ongoing innovations and pilots in practices, described as ‘pilotitis’,^43^ may be a distraction or even a barrier to implementing social prescribing.

### Implications for research and practice

Social prescribing with CLPs attached to GP practices is being widely advocated by policymakers as a means of reducing health inequalities, and large-scale roll-out is underway in the UK.^18^^–^22 The limited evidence base for this approach makes such policies questionable. The findings from this current study highlight the challenges in fully implementing a social prescribing approach within general practice, even in a well-supported and generously funded programme. Practice buy-in at an early stage, collaborative leadership, good team dynamics, and effective project management appear to be essential elements, and practices that can ensure these attributes may be in the minority. As this evaluation was only funded to commence 1 year after the LWP started, the authors cannot comment on how long embedding took in the FIPs, other than to note that differences between PIPs and FIPs were apparent early on in this evaluation. It is not possible to say whether the poorer implementation in the PIPs was because of systematic issues relating to the general style of the practices or was specific to the LWP.

In conclusion, health inequalities persist because of structural issues relating to the wider social determinants of health. Social prescribing (if effectively implemented), however, may help mitigate the effects of health inequalities. Nonetheless, in a well-resourced government-funded programme, the majority of practices in the current study had not fully integrated the LWP within the first 2 years. Implementing social prescribing and link workers within primary care at scale is unlikely to be a ‘quick fix’ for mitigating health inequalities in deprived areas.
